# Actualización en el diagnóstico bioquímico de la enfermedad de Wilson

**DOI:** 10.1515/almed-2021-0089

**Published:** 2022-05-09

**Authors:** Eduardo Martínez-Morillo, Josep Miquel Bauça, en el nombre de la Comisión de Elementos Traza de la Sociedad Española de Medicina de Laboratorio

**Affiliations:** Servicio de Análisis Clínicos y Bioquímica Clínica, Complejo Asistencial Universitario de Salamanca (CAUSA), Salamanca, España; Servicio de Análisis Clínicos, Hospital Universitario Son Espases, Palma de Mallorca, España

**Keywords:** ceruloplasmina, cobre, enfermedad de Wilson, gen *ATP7B*

## Abstract

La enfermedad de Wilson (EW) es un trastorno hereditario del metabolismo del cobre causado por mutaciones en el gen *ATP7B*, en el que se produce la acumulación de este elemento en el hígado y otros órganos y tejidos de los pacientes afectados, provocando principalmente manifestaciones hepáticas y neuropsiquiátricas. Se revisan el rendimiento diagnóstico y las limitaciones de las principales pruebas bioquímicas utilizadas en esta enfermedad infradiagnosticada. Además, se incluyen recomendaciones y se proponen comentarios estandarizados que podrían acompañar a los resultados en el informe de laboratorio. A pesar de no disponer aún de una prueba bioquímica rápida, sencilla y fiable que permita un diagnóstico inequívoco de la EW, la combinación de ceruloplasmina sérica y cobre urinario permite una orientación diagnóstica certera. El cobre sérico total debe ser utilizado con precaución dado su bajo valor predictivo negativo y no se recomienda el uso del cobre no unido a ceruloplasmina calculado. Sin embargo, el cobre intercambiable relativo medido presenta una sensibilidad y especificidad muy elevadas, pudiendo convertirse en un futuro en la prueba de referencia para el diagnóstico bioquímico de la EW. El desarrollo de nuevos métodos abre la puerta a la incorporación de la EW a los programas de cribado neonatal.

## Introducción

La enfermedad de Wilson (EW) es un trastorno hereditario autosómico recesivo del metabolismo del cobre, causado por mutaciones en el gen *ATP7B,* localizado en el brazo largo del cromosoma 13 (locus 13q14.3) [1, 2]. Este gen codifica para la síntesis de una ATPasa transportadora de cobre, que se expresa mayoritariamente en el hígado y que presenta seis dominios de unión a metales y ocho dominios transmembrana, los cuales forman un poro para el transporte ATP-dependiente del cobre a través de las membranas [3]. Esta proteína es esencial para la movilización de este elemento traza y su eliminación por excreción biliar [4].

En los pacientes con la EW, el cobre que se encuentra dentro de los hepatocitos no puede ser eliminado ni incorporado a la ceruloplasmina, la principal proteína transportadora de cobre, para su posterior secreción en el torrente sanguíneo [3]. Esto conduce a concentraciones excesivamente elevadas que se acumulan inicialmente en los hepatocitos y posteriormente en otros órganos y tejidos, particularmente el cerebro, provocando disfunción mitocondrial y apoptosis. Además, la formación de especies reactivas de oxígeno y la interacción directa del cobre en la síntesis lipídica provocan alteraciones en el metabolismo energético celular y la desregulación de genes involucrados en la biosíntesis de colesterol [4]. Por otro lado, las neuronas, especialmente las localizadas en los ganglios basales, son células particularmente vulnerables a los efectos provocados por el exceso de cobre. La consecuencia de todo esto es la presencia de una gran variedad de síntomas, incluyendo manifestaciones hepáticas, neuropsiquiátricas y oftalmológicas [3].

La mayoría de los pacientes con la EW son diagnosticados entre los 5 y 35 años, aunque la enfermedad puede hacerse sintomática a cualquier edad [5], habiéndose descrito casos de pacientes diagnosticados entre los 9 meses y los 77 años [6, 7]. Las manifestaciones hepáticas pueden ser extremadamente variables, desde hepatomegalia asintomática, esplenomegalia aislada, elevación intermitente o persistente de las transaminasas, ictericia, hígado graso, hepatitis aguda compensada o cirrosis descompensada, hasta fallo hepático agudo. Los síntomas neurológicos más frecuentes son disartria, temblores posturales, distonía, parkinsonismo, ataxia y corea. Aproximadamente 2/3 de los pacientes con EW presentan síntomas psiquiátricos en el momento del diagnóstico y hasta un 20% antes del mismo. Las características psiquiátricas más frecuentes son: comportamiento incongruente o antisocial, irritabilidad, cambios de personalidad y depresión. En cuanto a las manifestaciones oftalmológicas, los anillos de Kayser-Fleischer se presentan en la mayoría de los casos de EW con presentación neurológica, pero únicamente son observados en el 50% de los pacientes con presentación hepática y en menos del 30% de los pacientes asintomáticos [3, 8]. Otras manifestaciones clínicas menos frecuentes son: hematológicas (anemia hemolítica no autoinmune, coagulopatía, trombocitopenia), renales (fallo renal agudo, nefrolitiasis, urolitiasis, acidosis tubular renal), musculoesqueléticas (artropatía, debilidad muscular) u otras (enfermedad cardiaca, pancreatitis, hipoparatiroidismo) [5].

La prevalencia global estimada de la EW es de entre 1:10,000 y 1:30,000, con una frecuencia de portadores heterocigotos de 1:70 [9, 10]. Hasta la fecha, se han identificado más de 700 mutaciones patogénicas o probablemente patogénicas en el gen *ATP7B*, incluyendo 279 variantes asociadas a pérdida de función de la proteína [10]. Múltiples estudios han demostrado una mala correlación entre el genotipo y el fenotipo de la enfermedad, con una penetrancia incompleta y muy variable, pudiendo existir interacciones epigenéticas y factores metabólicos que contribuyen a la existencia de una gran variedad de fenotipos [11]. Además, las distintas variantes provocan diferentes grados de afectación en la homeostasis del cobre, estando las variantes con pérdida de función de la proteína asociadas a una elevada penetrancia y las variantes con cambio de sentido asociadas a una penetrancia menor. Por ello, para el diagnóstico genético de la EW es importante que las dos mutaciones detectadas sean patogénicas y, en todo caso, serán necesarias otras pruebas bioquímicas que permitan determinar el grado de disfunción de la homeostasis del cobre para establecer la necesidad o no de tratamiento [10, 12]. La metodología habitualmente utilizada para el diagnóstico molecular de la EW es la secuenciación completa del gen *ATP7B* para la detección de variantes patogénicas, ya sea por el método de referencia (secuenciación de Sanger) o mediante secuenciación masiva paralela (Next-Generation Sequencing, NGS) [13]. La técnica MLPA (Multiple Ligation-dependent Probe Amplification) puede usarse como metodología complementaria a la secuenciación directa para detectar grandes deleciones y duplicaciones. La principal ventaja de este estudio es que permite una confirmación diagnóstica de la enfermedad cuando se detectan dos variantes patogénicas asociadas a pérdida de función de la proteína, mientras que sus principales limitaciones son que se trata de un estudio relativamente caro y la dificultad de interpretación de algunos casos donde se identifican variantes de significado clínico incierto [14]. Es por ello que el diagnóstico molecular suele delimitarse a los casos altamente sospechosos de EW, ya sea por hallazgos clínicos o bioquímicos.

Un diagnóstico temprano es esencial para prevenir complicaciones a largo plazo. Actualmente, se sigue considerando que la EW es una patología infradiagnosticada, siendo el diagnóstico tardío la causa más frecuente de consecuencias graves y muerte [15]. Por el contrario, el tratamiento precoz de los pacientes asintomáticos puede prevenir la aparición de las manifestaciones clínicas [4]. Sin embargo, hasta el momento, la mayoría de los pacientes asintomáticos son diagnosticados durante cribados genéticos familiares realizados tras haberse identificado un caso en un miembro de la unidad familiar [5].

El diagnóstico de la EW sigue siendo complicado por no disponer de una prueba rápida, sencilla, fiable y certera que sea patognomónica de la enfermedad. En promedio, el tiempo transcurrido entre la aparición de los primeros síntomas y el diagnóstico es superior a los dos años, siendo los pacientes con presentación neurológica los que reciben un diagnóstico más tardío [8]. Habitualmente, los pacientes con la EW tienen unas características clínicas inespecíficas cuyo diagnóstico se consigue tras la realización de una combinación de pruebas clínicas y de laboratorio [16]. Además, la existencia de casos de presentación atípica dificulta aún más el diagnóstico [8]. A esto hay que añadir que una mala interpretación de los síntomas y/o de las pruebas de laboratorio puede llevar a establecer erróneamente el diagnóstico de EW, con el consiguiente tratamiento innecesario y sus posibles efectos secundarios [17].

Actualmente, la mayoría de los tratamientos en Europa para la EW incluyen el uso de la D-penicilamina, la trientina o las sales de zinc como principales opciones terapéuticas. Los dos primeros son agentes quelantes utilizados para capturar el exceso de cobre corporal y aumentar su eliminación a través de la orina, mientras que las sales de zinc inhiben la absorción intestinal del cobre procedente de la dieta. De todos estos tratamientos, la D-penicilamina es la que se ha asociado con un mayor número de eventos adversos [18]. Finalmente, en los casos más graves de la EW puede ser necesario recurrir a un trasplante hepático [3, 5].

## Objeto y campo de aplicación

El presente documento tiene por objeto revisar las principales pruebas bioquímicas existentes para el diagnóstico de la EW, mediante la recopilación de la evidencia científica disponible con respecto a su rendimiento diagnóstico, sus limitaciones y las principales causas de resultados falsos positivos (FPs) y falsos negativos (FNs). Todo ello para que pueda ser utilizado como una fuente de consulta rápida que ayude a interpretar adecuadamente las pruebas y sus resultados y así llegar a un diagnóstico más preciso de la EW. Además, se incluyen unas breves recomendaciones y se proponen varios comentarios estandarizados que podrían acompañar a los resultados en el informe de laboratorio (Tablas 1 y 2).

**Tabla 1:** Recomendaciones para el análisis e interpretación de los resultados de ceruloplasmina y cobre en el diagnóstico de la enfermedad de Wilson (EW).

	Recomendación
**Fase pre-analítica**	
Ceruloplasmina	No se debe cuantificar esta proteína en muestras muy lipémicas.
	Se pueden utilizar muestras tanto de suero como de plasma, aunque se recomienda el uso del suero si se va a realizar la determinación simultánea de cobre.
Cobre en sangre	Las muestras de plasma no son adecuadas para la estimación del estado nutricional del cobre ni para el diagnóstico de la EW.
	La concentración de cobre sérico total tiene un valor predictivo negativo muy bajo para la EW y, por lo tanto, no debe ser utilizada para descartar esta enfermedad.
Cobre urinario	Se recomienda la utilización de una orina de 24 horas, no existiendo evidencia científica suficiente que avale el uso de la orina de micción aislada para el diagnóstico de la EW.
Cobre hepático	No se debe realizar la cuantificación del cobre intrahepático en pacientes con un proceso colestásico (como una colangitis biliar primaria o una colangitis esclerosante primaria).
**Fase post-analítica**	
Ceruloplasmina	Ante la sospecha de un proceso de fase aguda (por ejemplo, inflamación) se recomienda determinar la concentración de otro reactante de fase aguda (como la proteína C reactiva) para identificar posibles falsos negativos.
Cobre sérico	Se recomienda añadir la determinación de ceruloplasmina ante un hallazgo aislado de una concentración de cobre sérico reducida en pacientes jóvenes o sin causa que justifique un déficit de cobre.
	Se debe descartar la contaminación de la muestra ante concentraciones de cobre sérico superiores a 8 μmol/dL (500 μg/dL).
Cobre urinario	Se deben interpretar con precaución los resultados del cobre urinario en pacientes con daño renal, ya que existe una asociación significativa entre un filtrado glomerular disminuido y una mayor excreción urinaria de cobre, no estando esta prueba indicada en los casos más graves de insuficiencia renal por el elevado riesgo de obtener resultados falsos positivos.
**Marcadores bioquímicos**	
Cobre no unido a ceruloplasmina	No se recomienda el uso de fórmulas para el cálculo del cobre no unido a ceruloplasmina.
Cobre intercambiable relativo	Este marcador bioquímico tiene un gran potencial diagnóstico y debería ser evaluado en los laboratorios clínicos.

**Tabla 2:** Comentarios estandarizados para el informe de resultados de ceruloplasmina y cobre en el diagnóstico de la enfermedad de Wilson (EW).

	Comentario
**Fase pre-analítica**	
Muestra de suero muy lipémica	No se puede realizar la determinación de ceruloplasmina en muestras altamente lipémicas.
**Fase post-analítica**	
Ceruloplasmina: <0,1 g/L	Las concentraciones de ceruloplasmina y cobre urinario son altamente sugestivas de EW.
Cobre urinario: >1,6 µmol/24 h	
	
Ceruloplasmina: >0,2 g/L	Las concentraciones de ceruloplasmina y cobre urinario indican que un diagnóstico de EW es improbable.
Cobre urinario: <0,64 µmol/24 h (40 µg/24 h)	
	
Cobre sérico total: <0,8 μmol/dL (50 μg/dL)	Se recomienda descartar un déficit grave de cobre o la EW.
	
Cobre sérico total: >4 μmol/dL (250 μg/dL)	Se recomienda descartar un fallo hepático fulminante (no aplica en embarazadas y mujeres en tratamiento con estrógenos).
	
Cobre sérico total: >8 μmol/dL (500 μg/dL)	Se recomienda enviar una nueva muestra para descartar una contaminación por cobre.

## Pruebas bioquímicas para el diagnóstico de la EW

En condiciones ideales, se debería disponer de una prueba rápida, fiable, no invasiva y con poder discriminatorio que permita un diagnóstico certero de la EW. Sin embargo, la mayoría de las pruebas existentes presentan limitaciones y de forma aislada son insuficientes para poder diagnosticar eficazmente la enfermedad. Por ello, es fundamental realizar una interpretación adecuada de los resultados para llegar a un diagnóstico correcto.

En general, la EW se caracteriza bioquímicamente por una concentración disminuida de ceruloplasmina y cobre total en suero, una excreción aumentada de cobre urinario, y un contenido de cobre intrahepático anormalmente elevado [1]. Sin embargo, la triada clásica: ceruloplasmina baja, cobre sérico bajo y cobre urinario elevado puede estar ausente en un número significativo de casos de EW y estar presente en más del 15% de portadores heterocigotos [8].

### Ceruloplasmina sérica

La ceruloplasmina, una enzima con actividad ferroxidasa, es la principal proteína encargada del transporte del cobre, conteniendo hasta un 70–90% del cobre sérico circulante. Es sintetizada en el hígado en forma de apoceruloplasmina, enzimáticamente inactiva, donde une de seis a ocho átomos de cobre y se activa, pasando a llamarse holoceruloplasmina. La captura de cobre por parte de la apoceruloplasmina y su posterior liberación a la circulación por los hepatocitos es dependiente de la proteína ATP7B. Si esta unión de cobre no se produce, la apoceruloplasmina es degradada rápidamente. Es por ello que una concentración sérica baja de holoceruloplasmina es una característica bioquímica muy destacada de la EW [4, 5].

La ceruloplasmina suele medirse en suero (aunque también se puede medir en plasma) mediante métodos inmunoturbidimétricos o inmunonefelométricos, los cuales cuantifican tanto la holoceruloplasmina como la apoceruloplasmina, como ayuda en el diagnóstico de la EW o para la identificación de otras condiciones asociadas a un déficit de cobre. Una revisión reciente muestra a través de un metaanálisis que la ceruloplasmina tiene una sensibilidad que va del 77 al 99% y una especificidad que va del 56 al 83% para el diagnóstico de la EW (según los diferentes estudios incluidos), cuando se usa un punto de corte de 0,2 g/L (límite inferior de referencia (LIR) establecido durante el International Meeting on Wilson’s disease, en Leipzig 2001 [19] y declarado actualmente por la mayoría de los fabricantes). Si se baja el punto de corte a 0,1 g/L, la sensibilidad disminuye hasta entre un 65 y 79%, pero la especificidad aumenta hasta entre un 97 y 100% [16]. Dada la falta de estandarización de los métodos que miden ceruloplasmina, es importante tener en cuenta el LIR declarado por el fabricante, ya que si este es diferente a 0,2 g/L (como es el caso del reactivo CERU para Cobas^®^ (Roche Diagnostics)), estos puntos de corte podrían generar un rendimiento diagnóstico diferente.

La concentración de ceruloplasmina puede elevarse durante el embarazo o con el uso de anticonceptivos y el tratamiento con estrógenos. Además, al ser un reactante de fase aguda positivo, también se puede elevar en casos de inflamación, infección, en la artritis reumatoide y en pacientes con complicaciones miocárdicas o cáncer (FNs). Por el contrario, se puede obtener una concentración sérica baja (FPs) en casos de hepatitis aguda viral, enfermedad hepática inducida por drogas, alcohólica o terminal, malabsorción, malnutrición, caquexia, nefropatía con pérdida proteica, deficiencia adquirida de cobre (por ejemplo, tras una intoxicación por zinc), enfermedad de Menkes, aceruloplasminemia o en portadores sanos (Tabla 3) [5, 8].

**Tabla 3:** Pruebas bioquímicas, rendimiento diagnóstico y causas de resultados falsos positivos (FPs) y negativos (FNs) en la EW.

Prueba	Punto de corte	Sensibilidad	Especificidad	Causas de FPs	Causas de FNs	Referencias
Ceruloplasmina	<0,1 g/L	65–79%	97–100%	Hepatitis aguda viral; enfermedad hepática inducida por drogas; enfermedad hepática alcohólica; enfermedad hepática terminal; malabsorción; malnutrición; caquexia; nefropatía con pérdida proteica; deficiencia adquirida de cobre; enfermedad de Menkes; aceruloplasminemia; portadores heterocigotos; muestras lipémicas	Embarazo; anticonceptivos; estrógenos; inflamación; infección; artritis reumatoide; daño miocárdico; cáncer; método inmunoquímico	[16]
	<0,2 g/L	77–99%	56–83%			
Cobre urinario	>0,64 µmol/24 h (40 µg/24 h)	79%	88%	Hepatitis autoinmune; enfermedad hepática crónica activa; colestasis; fallo hepático agudo; portadores heterocigotos	Fallo renal; recolección inadecuada o incompleta de la muestra	[35]
	>1,6 µmol/24 h (100 µg/24 h)	50–80%	76–97%			[16]
Cobre hepático	>4 μmol/g (250 μg/g)	66–94%	52–99%	Colestasis	Distribución heterogénea de los depósitos	[16]
Cobre intercambiable relativo	14-18,5%	92–100%	99–100%	No se han descrito		[29, 32, 47]

No se incluyen en la Tabla ni el cobre sérico total ni el cobre no unido a ceruloplasmina calculado por no estar recomendado su uso en el diagnóstico de la enfermedad de Wilson (EW).

Una limitación analítica de la medición de ceruloplasmina es el uso generalizado de métodos inmunoquímicos para su determinación. Estos métodos, al medir la concentración tanto de la holoceruloplasmina como de la apoceruloplasmina, sobreestiman de forma indirecta la actividad enzimática de la holoceruloplasmina, cuando se comparan los resultados con los obtenidos con métodos basados en la medición directa de la actividad enzimática [8]. Por lo tanto, concentraciones normales de ceruloplasmina no descartan la posibilidad de una baja actividad ferroxidasa (FN). La actividad enzimática específica de la ceruloplasmina es sensible al estado del cobre y no se ve afectada por la edad, sexo o alteraciones hormonales [20]. Por ello, se considera más adecuado el uso de métodos enzimáticos para el diagnóstico de la EW, aunque estos no suelen estar disponibles en los laboratorios clínicos, sobre todo como consecuencia de la inestabilidad de las muestras y de la falta de estandarización [4, 5, 21]. Por último, las muestras altamente lipémicas deben ser evitadas ya que su análisis puede generar resultados falsamente disminuidos (FP) [17]. Esto no suele indicarse en la ficha técnica del fabricante debido a que los estudios de interferencias se realizan habitualmente con Intralipid^®^ y esta emulsión no siempre es representativa de la composición tan compleja que tienen muchas muestras de pacientes con altas concentraciones de triglicéridos [22, 23].

### Cobre sérico

El cobre es un elemento traza necesario como cofactor para el adecuado funcionamiento de múltiples enzimas, siendo el suero la muestra de elección para valorar el estado nutricional del cobre en el organismo. No se recomienda la utilización de muestras de plasma, ya que la concentración es inferior a la del suero y parece no reflejar adecuadamente el estado nutricional de cobre [24]. Su determinación tanto en suero como en orina se realiza principalmente mediante la espectrometría de masas con plasma de acoplamiento inductivo (ICP-MS) o la espectrometría de absorción atómica, siendo actualmente la ICP-MS la tecnología más utilizada por los participantes del Programa Europeo de Evaluación Externa de la Calidad OELM. Ambas metodologías requieren de una serie de consideraciones preanalíticas, técnicas y de preparación de muestras que han sido discutidas en detalle en un documento previo de la comisión de elementos traza [25, 26].

En condiciones normales, la concentración sérica de cobre varía proporcionalmente con la concentración de ceruloplasmina. Por ello, habitualmente se encuentra reducido en la EW. Sin embargo, es frecuente observar en algunos de estos pacientes concentraciones normales o incluso elevadas, lo cual suele ser indicativo de la presencia de una gran cantidad de cobre circulante no unido a ceruloplasmina [5]. Existen varias condiciones clínicas que pueden provocar esta elevación de la concentración del cobre sérico independientemente de la concentración de ceruloplasmina como, por ejemplo, un fallo hepático agudo de cualquier etiología, debido a la liberación profusa de cobre procedente del tejido hepático, la colestasis crónica o, menos frecuentemente, una intoxicación por cobre (FNs). Elevaciones de la concentración de cobre en mujeres en edad fértil suelen estar relacionadas con el embarazo o terapias con anticonceptivos orales o estrógenos. Por otro lado, y aunque es menos común, pueden obtenerse resultados de cobre disminuidos (FPs) en pacientes con un déficit adquirido (por cirugía de bypass gástrico, gastrectomía o una ingesta excesiva de zinc, entre otras causas), en la enfermedad de Menkes y en un pequeño porcentaje de portadores heterocigotos [4, 27].

A pesar de ser una práctica clínica habitual, varias asociaciones de ámbito nacional e internacional (como EASL, AASLD, AEEH o APASL), no recomiendan la utilización del cobre sérico total para el diagnóstico de la EW, fundamentalmente por su bajo valor predictivo negativo, presentando una especificidad que va del 94 al 100% pero una sensibilidad que va del 69 al 74%, según los estudios consultados [27], [28], [29]. Por ello, una concentración normal o elevada de cobre sérico no permite descartar la enfermedad. Sin embargo, una concentración anormalmente baja es altamente sugestiva de EW, dado el elevado valor predictivo positivo de la prueba, una vez descartadas otras causas de déficit de cobre. Por ello, ante un hallazgo aislado de un resultado de cobre disminuido, especialmente en pacientes jóvenes, estaría recomendada la determinación de ceruloplasmina sérica.

El intervalo de referencia definido para el cobre sérico es de 1,1–2,5 μmol/dL (70–155 μg/dL) [25], existiendo pequeñas variaciones en función de la edad y el sexo y en especial en mujeres embarazadas o en tratamiento con estrógenos, las cuales pueden presentar concentraciones de hasta 4,75 μmol/dL (300 μg/dL) [20]. Concentraciones por debajo de 0,8 μmol/dL (50 μg/dL) requieren descartar un déficit grave de cobre o la EW [29], [30], [31], [32]. Concentraciones por encima de 4 μmol/dL (250 μg/dL) son poco frecuentes y pueden indicar una insuficiencia hepática fulminante [28, 33]. Valores por encima de 8 μmol/dL (500 μg/dL) son muy raros y requieren que se descarte una contaminación de la muestra.

### Cobre urinario

La imposibilidad de eliminar el cobre por excreción biliar provoca que este sea eliminado por la orina en los pacientes con la EW, aumentando así su concentración. A pesar de no existir un punto de corte claramente definido, se recomienda utilizar un valor más bajo en población pediátrica que en población adulta debido al menor tiempo de acumulación del cobre en el organismo [34].

El cobre en orina de 24 horas tiene una sensibilidad del 79% y una especificidad del 88% para el diagnóstico de la EW, si se usa un punto de corte de 0,64 µmol/24 h (40 µg/24 h), y una sensibilidad que va del 50 al 80% y una especificidad que va del 76 al 97%, si se usa un punto de corte de 1,6 µmol/24 h (100 µg/24 h), según los estudios consultados para población pediátrica [16, 35]. A pesar de los inconvenientes que implica la recogida de una orina de 24 horas, no se recomienda el estudio del cobre en muestras de orina de micción aislada al presentar una gran variabilidad intraindividual [25]. Además, aunque se ha evaluado la posibilidad de corregir la cantidad de cobre por la concentración de creatinina o incluso de zinc, no existe suficiente evidencia que lo avale ni valores de referencia claramente establecidos [36, 37].

La interpretación de los resultados en orina puede ser complicada en algunos casos, especialmente cuando el paciente presenta daño renal, ya que los pacientes con un filtrado glomerular disminuido presentan mayores concentraciones de cobre urinario [38, 39], o cuando se realiza una recolección de la muestra inadecuada o incompleta (FNs). Además, se pueden observar valores elevados de cobre urinario en otras enfermedades hepáticas (FPs): hepatitis autoinmune, enfermedad hepática crónica activa o síndromes colestásicos y particularmente durante un fallo hepático agudo de cualquier tipo. Los portadores heterocigotos también suelen presentar concentraciones de cobre urinario intermedias entre las observadas en los pacientes con la EW y los pacientes sin la enfermedad, pudiendo en algunos casos llegar a superar el límite superior de referencia (LSR) (Tabla 3) [5, 8].

### Cobre hepático

La biopsia hepática es una técnica invasiva que, al no estar libre de riesgos, no se suele realizar en pacientes asintomáticos ni pediátricos. Su uso se suele limitar a pacientes adultos con sospecha clínica o bioquímica de padecer la EW, generalmente con una presentación hepática, pero sin un diagnóstico definitivo. Raramente es necesario recurrir a una biopsia hepática en los pacientes con una presentación neuropsiquiátrica de la enfermedad [3, 5].

El tratamiento de la muestra ha de realizarse en unas condiciones concretas según se indica en la guía del Clinical and Laboratory Standards Institute (CLSI), siendo fundamental obtener una cantidad adecuada de tejido (normalmente el tamaño mínimo es de entre 5 y 10 mm), que debe ser lavado con agua desionizada (nunca usar solución salina) e introducido en un tubo de polipropileno sin añadir agua ni ningún otro líquido. El recipiente debe ser enviado perfectamente cerrado al laboratorio en las primeras 24 horas tras su recogida y, si no es posible, se debe congelar la muestra a −20 °C [40].

El cobre en tejido hepático tiene una sensibilidad que va del 66 al 94% y una especificidad que va del 52 al 99% para el diagnóstico de la EW, según los estudios consultados, si se usa un punto de corte de 4 μmol/g peso seco (250 μg/g). Las principales limitaciones de su cuantificación son la heterogeneidad en la distribución de los depósitos de cobre intrahepáticos, lo cual puede generar resultados FNs si la muestra obtenida no es representativa, así como la elevación de estos depósitos en pacientes con enfermedad colestásica (FPs) (Tabla 3). Por ello, algunos aspectos como el tamaño de muestra necesario y el punto de corte óptimo siguen siendo, hoy en día, un tema de debate [5, 16, 41].

### Cobre sérico no unido a ceruloplasmina (NCC)

El cobre sérico no unido a ceruloplasmina (NCC) calculado fue propuesto como marcador diagnóstico de la EW, ya que en teoría debe encontrarse elevado en el suero de los pacientes con esta enfermedad. Su concentración se puede obtener mediante la siguiente fórmula:

NCC (μmol/L)= Cobre total (μmol/L)−49 (μmol/g)× Ceruloplasmina(g/L)

Siendo 49 el factor que permite estimar la cantidad de cobre (en µmol/L) unido a ceruloplasmina, según la concentración sérica (en g/L) de esta proteína.

La utilidad de este marcador ha sido ampliamente debatida, publicándose estudios en los que se ha encontrado hasta un 25% de resultados FNs en pacientes con la EW, si se usa un método inmunoquímico para la determinación de la ceruloplasmina. Además, el NCC puede encontrarse elevado (FPs) en síndromes colestásicos, fallo hepático agudo y en pacientes con intoxicación por cobre [5]. Por otro lado, se ha descrito que el cálculo de este parámetro no es transferible entre laboratorios y que se pueden obtener resultados inferiores a cero en más del 20% de individuos, lo cual no es fisiológicamente posible. Por todo ello, y al igual que ocurre con el cobre sérico, la Asociación Europea para el Estudio del Hígado (EASL) no recomienda su uso para el diagnóstico de la EW [27, 42, 43].

### Cobre intercambiable (CuEXC) y cobre intercambiable relativo (REC)

En los últimos años se han publicado varios estudios en los que se evalúa el rendimiento diagnóstico de la medición directa del cobre intercambiable (CuEXC) en muestras de suero de pacientes con la EW, habiéndose obtenido resultados muy prometedores en cuanto a sensibilidad y especificidad [8].

Entre el 70 y 90% del cobre circulante se encuentra unido a ceruloplasmina, existiendo una pequeña proporción unida a albúmina (≈15%), α2-macroglobulina (≈10%), aminoácidos o bien circulando libremente (<5%). Así, se cree que el CuEXC representa el cobre libre y el cobre unido de forma lábil principalmente a la albúmina, pero también a la α2-macroglobulina, aminoácidos y a la estructura externa de la ceruloplasmina, representando el cobre biodisponible para la mayoría de las células gracias a esta unión más lábil [29, 44, 45].

La determinación del CuEXC requiere un sencillo proceso de incubación de las muestras de suero, durante 1 hora, con una solución de EDTA y la posterior ultrafiltración del suero diluido [29]. Sin embargo, el marcador que ha mostrado el mayor potencial diagnóstico para la EW es el cobre intercambiable relativo (REC):

REC (%)=(CuEXC/Cu total)×100

Siendo la concentración de Cu total la obtenida en la muestra de suero original y la concentración de CuEXC la obtenida en el ultrafiltrado tras el procesamiento de la muestra.

El Balkhi y col. encontraron que el REC presentaba un rendimiento diagnóstico del 100% en la detección de 16 casos de EW (punto de corte: >18,5%), cuando se compararon los resultados obtenidos con los de 62 individuos sanos, 25 familiares de los pacientes con la EW y sin mutaciones en el gen *ATP7B* y 45 portadores heterocigotos [29]. En un estudio posterior, Trocello y col. comprobaron que el REC también permite discriminar entre pacientes con la EW (homocigotos o doble heterocigotos) y portadores heterocigotos. Todos los pacientes con la EW mostraron un REC >15%, mientras que ninguno de los demás participantes superó este punto de corte [32]. Poujois y col. también evaluaron la concentración del CuEXC en pacientes con la EW y distinto tipo de presentación clínica: pre-sintomáticos, con presentación hepática y con presentación extra-hepática, observando que el CuEXC se encontraba significativamente más elevado en los pacientes con presentación extra-hepática, lo que sugiere que podría ser un indicador de gravedad [46].

REC también ha mostrado un rendimiento diagnóstico muy elevado al comparar los resultados obtenidos en pacientes con la EW y con otras enfermedades hepáticas, con una sensibilidad del 100% y una especificidad del 99%, usando un punto de corte del 14% y una sensibilidad del 92% (no detectados 4 casos de EW a tratamiento) y una especificidad del 100% al usar un punto de corte del 18,5% [47].

Por lo tanto, CuEXC y REC parecen proporcionar información sobre la gravedad y el grado de extensión de la EW, además de un gran potencial diagnóstico. A esto hay que añadir que recientemente se han publicado valores de referencia tanto para el CuEXC como para el REC en población de 1–18 años [48, 49]. No obstante, son necesarios estudios prospectivos que permitan confirmar la fiabilidad de este parámetro bioquímico, aunque los resultados obtenidos hasta el momento alientan a usar esta prueba en casos seleccionados para, por ejemplo, determinar que pacientes podrían beneficiarse de un estudio molecular de gen *ATP7B*, ya que aunque el análisis del CuEXC implica un incremento en el coste de cada determinación de aproximadamente unos 10 euros (con un coste total no superior a los 20 euros), es muy inferior al coste de un análisis genético, el cual supone entre 500 y 1,000 euros.

### Otras pruebas bioquímicas

Dado que los pacientes con un fallo hepático fulminante provocado por la EW suelen presentar resultados FNs de ceruloplasmina y cobre sérico total, lo cual dificulta el diagnóstico de la enfermedad, otros parámetros bioquímicos clásicos han sido evaluados para tratar de identificar a estos pacientes. Así, Korman y col. describieron que, en combinación con otros síntomas hallados en el paciente, un ratio fosfatasa alcalina (ALP)/bilirrubina total <4 presenta una sensibilidad del 94% y una especificidad del 96% para el diagnóstico de la EW, mientras que un ratio AST/ALT>2,2 presenta una sensibilidad del 94% y una especificidad del 86% [28]. Más recientemente, se ha propuesto un sistema de puntuación basado en los resultados de AST, ALT, ALP, ratio AST/ALT, urato y hemoglobina que permite obtener una sensibilidad del 88% y una especificidad del 87% para el diagnóstico de la EW fulminante [33]. Sin embargo, el rendimiento diagnóstico de estas pruebas parece ser bastante inferior en población pediátrica [50].

## Criterios diagnósticos de la EW

Como se ha visto, con la excepción del CuEXC y el REC (que aún se encuentran en investigación), ninguna de las pruebas bioquímicas utilizadas de forma rutinaria permite un diagnóstico certero de la EW. Por ello, varias sociedades han publicado guías clínicas con diferentes criterios diagnósticos. En concreto, la Asociación Americana para el Estudio de las Enfermedades Hepáticas (AASLD) propone un algoritmo clínico/bioquímico para el diagnóstico de la enfermedad, mientras que la EASL y la Sociedad Europea de Gastroenterología, Hepatología y Nutrición Pediátrica (ESPGHAN) se muestran a favor de usar la puntuación de Leipzig (Tabla 4) [19].

**Tabla 4:** Puntuación de Leipzig para el diagnóstico de la EW^a^.

Síntomas, signos y pruebas (puntuación)	
**Ceruloplasmina sérica**	**Anillos de Kayser-Fleischer**

>0,2 g/L: 0 puntos	Presentes: 2 puntos
0,1–0,2 g/L: 1 punto	Ausentes: 0 puntos
<0,1 g/L: 2 puntos	
**Cobre urinario** ^**b**^	**Síntomas neurológicos**

Normal: 0 puntos	Graves: 2 puntos
1–2 × LSR: 1 punto	Leves: 1 punto
>2 × LSR: 2 puntos	Ausentes: 0 puntos
**Cobre intrahepático** ^**c**^	**Análisis genético** ^**d**^

>4 μmol/g (>250 μg/g): 2 puntos	Mutación en dos alelos: 4 puntos
0,8–4 μmol/g (50–250 μg/g): 1 punto	Mutación en un alelo: 1 punto
<0,8 μmol/g (<50 μg/g): −1 punto	Ninguna mutación: 0 puntos
**Anemia hemolítica Coombs negativa**	**Puntuación final**

Presente: 1 punto	≥4 puntos, diagnóstico establecido
Ausente: 0 puntos	3 puntos, diagnóstico posible
	≤2 puntos, diagnóstico muy improbable

^a^Modificado de (19). ^b^En ausencia de hepatitis aguda; ^c^en ausencia de colestasis; ^d^detección de variantes clasificadas como patogénicas o probablemente patogénicas; EW, enfermedad de Wilson; LSR, límite superior de referencia.

El algoritmo propuesto por la AASLD se basa en un examen oftalmológico inicial para la búsqueda de los anillos de Kayser-Fleischer y en la determinación de la ceruloplasmina sérica y el cobre urinario, progresando este algoritmo de forma ligeramente diferente dependiendo de si el paciente presenta síntomas neuropsiquiátricos y/o hepáticos. En aquellos casos con resultados discordantes, se recomienda recurrir a la biopsia hepática o al análisis genético. Por el contrario, la estrategia diagnóstica defendida por la EASL y la ESPGHAN se basa en el sistema de puntuación de Leipzig, dándole un papel menos relevante para la confirmación diagnóstica a la biopsia hepática en favor del estudio genético, con el objetivo de evitar la realización de pruebas invasivas [19, 27, 51, 52]. Las principales limitaciones de este sistema de puntuación son que está basado en la opinión de expertos el lugar de en estudios poblacionales y la falta de consenso en la definición del LSR para la excreción de cobre urinario. No obstante, ha sido evaluado en población pediátrica con resultados satisfactorios, obteniéndose una sensibilidad que va del 93 al 98% y una especificidad que va del 92 al 97% [5, 35, 53].

**Figura 1:**
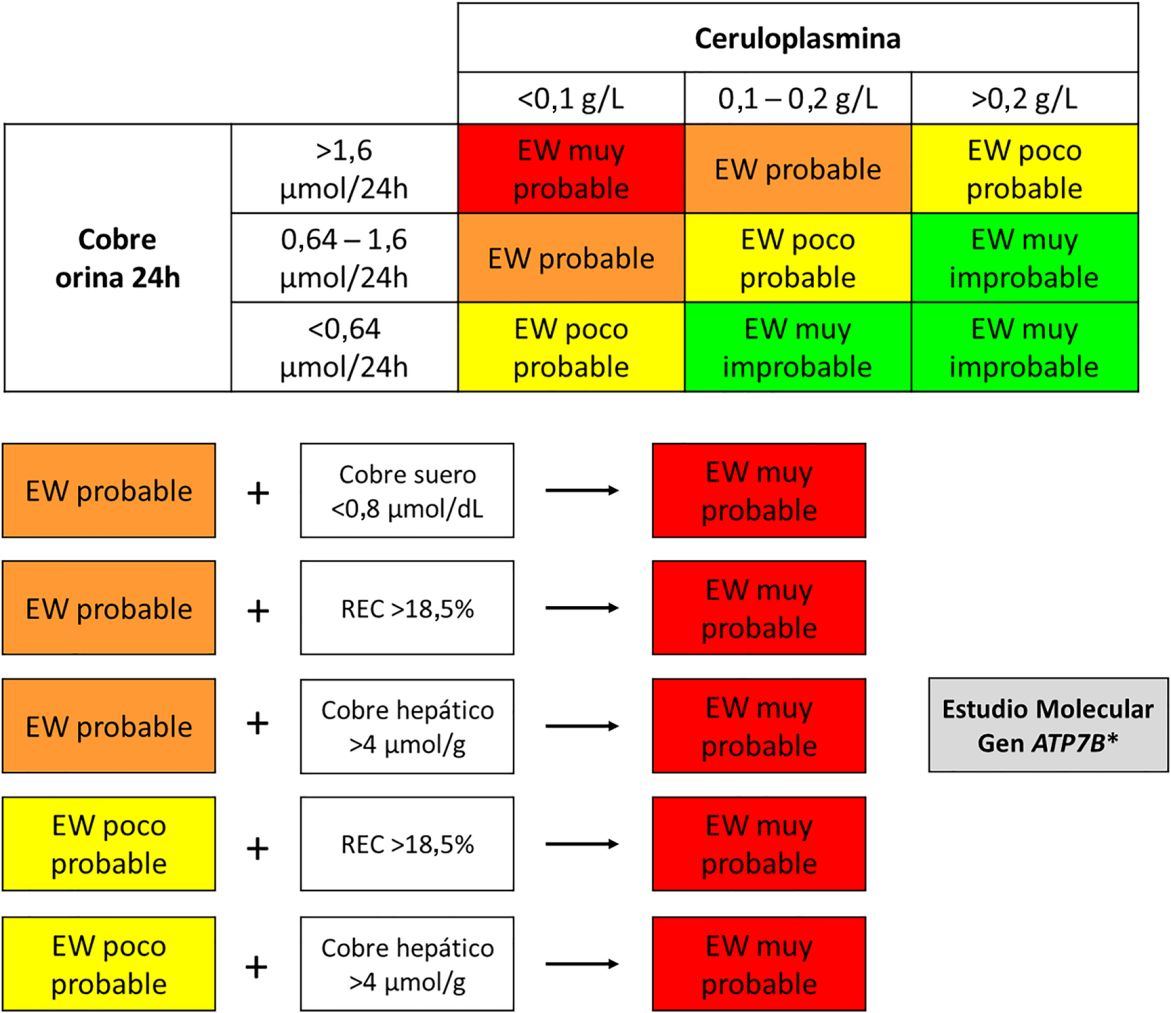
Algoritmo para el diagnóstico bioquímico de la enfermedad de Wilson (EW). Un resultado de cobre sérico total <0,8 μmol/dL puede ser utilizado para orientar los casos con resultados intermedios de ceruloplasmina hacia un diagnóstico muy probable de EW (gracias a su elevado valor predictivo positivo). Un resultado de cobre intercambiable relativo (REC)>18,5% o un resultados de cobre intrahepático >4 μmol/g, en ausencia de colestasis, permiten reorientar los casos dudosos hacia un diagnóstico muy probable de EW. *El estudio molecular está altamente indicado en los pacientes cuyos resultados indiquen una EW muy probable o en los pacientes con resultados que sugieran una EW probable y en los que se quiera evitar una biopsia hepática.

Teniendo en cuenta lo descrito anteriormente, en la Figura 1 se propone un algoritmo para establecer el diagnóstico bioquímico de la EW según los resultados obtenidos en las pruebas.

## Cribado neonatal de la EW

La EW cumple la mayoría de los criterios de Wilson and Jungner, los cuales son utilizados como referente para determinar que enfermedades se deben incorporar a los programas de cribado neonatal. Así, la EW puede considerarse como un problema importante de salud, con una historia natural conocida, un periodo de latencia generalmente largo donde la enfermedad no presenta síntomas, y con disponibilidad de un tratamiento eficaz que previene la aparición de las manifestaciones típicas. Sin embargo, si la EW aún no ha sido incorporada a los programas de cribado es porque no se dispone de una prueba o examen coste-efectivo que permita detectarla. Los estudios realizados con la ceruloplasmina como prueba de cribado han resultado poco satisfactorios, con un número significativo de resultados FNs, al tratarse de un reactante de fase aguda positivo, y resultados FPs, al existir un número no desdeñable de neonatos con concentraciones fisiológicamente bajas de ceruloplasmina [54], [55], [56], [57].

No obstante, recientemente se ha desarrollado un método en el que se miden, mediante espectrometría de masas en tándem, péptidos procedentes de la proteína ATP7B en muestras de sangre seca impregnada en papel (prueba del talón), tras un tratamiento de enriquecimiento de las muestras con anticuerpos. Este método tiene una sensibilidad del 91% y una especificidad del 98%, pudiendo representar el camino a seguir para poder incorporar la EW en los programas de cribado neonatal en un futuro cercano [58, 59].

## Conclusiones

Las pruebas bioquímicas habitualmente disponibles para el diagnóstico de la EW presentan limitaciones que hacen que el rendimiento sea subóptimo. Los datos disponibles sobre el CuEXC y el REC son prometedores y deben alentar a los especialistas de laboratorio a incorporar y evaluar estas pruebas en sus centros de trabajo ya que, aunque cada vez es mayor la disponibilidad de pruebas genéticas para la confirmación diagnóstica de la EW, la ausencia de correlación genotipo-fenotipo hace necesario disponer de otros marcadores que permitan estudiar adecuadamente el metabolismo del cobre en estos pacientes. Finalmente, el cribado neonatal de la EW podría convertirse en una realidad en un futuro próximo si se consigue desarrollar un método coste-efectivo.

## Anexo 1

Composición de la comisión (por orden alfabético): J.M. Bauça Rosselló, P. Bermejo Barrera, A. Bravo Gómez, J.A. Cocho de Juan, M. González Estecha (Presidenta desde marzo de 2019), J. González Revaldería, S. Izquierdo Álvarez, M.T. Llorente Ballesteros, E. Martínez González, E. Martínez Morillo, J.P. Sánchez Marín, S. Pérez San Martín y E. Urrechaga Igartua.
